# Yield of intermittent versus continuous EEG in comatose survivors of cardiac arrest treated with hypothermia

**DOI:** 10.1186/cc12879

**Published:** 2013-09-04

**Authors:** Vincent Alvarez, Alba Sierra-Marcos, Mauro Oddo, Andrea O Rossetti

**Affiliations:** 1 Department of Clinical Neurosciences, Centre Hospitalier Universitaire Vaudois, and University of Lausanne, Rue du Bugnon 46, CHUV BH-13, CH-1011 Lausanne, Switzerland; 2 Department of Intensive Care Medicine, Centre Hospitalier Universitaire Vaudois, and University of Lausanne, Rue du Bugnon 46, CHUV BH-13, CH-1011 Lausanne, Switzerland

## Abstract

**Introduction:**

Electroencephalography (EEG) has a central role in the outcome prognostication in subjects with anoxic/hypoxic encephalopathy following a cardiac arrest (CA). Continuous EEG monitoring (cEEG) has been consistently developed and studied; however, its yield as compared to repeated standard EEG (sEEG) is unknown.

**Methods:**

We studied a prospective cohort of comatose adults treated with therapeutic hypothermia (TH) after a CA. cEEG data regarding background activity and epileptiform components were compared to two 20-minute sEEGs extracted from the cEEG recording (one during TH, and one in early normothermia).

**Results:**

Thirty-four recordings were studied. During TH, the agreement between cEEG and sEEG was 97.1% (95% CI: 84.6 to 99.9%) for background discontinuity and reactivity evaluation, while it was 94.1% (95% CI 80.3 to 99.2%) regarding epileptiform activity. In early normothermia, we did not find any discrepancies. Thus, concordance results were very good during TH (kappa 0.83), and optimal during normothermia (kappa = 1). The median delay between CA and the first EEG reactivity testing was 18 hours (range: 4.75 to 25) for patients with perfect agreement and 10 hours (range: 5.75 to 10.5) for the three patients with discordant findings (*P *= 0.02, Wilcoxon).

**Conclusions:**

Standard intermittent EEG has comparable performance with continuous EEG both for variables important for outcome prognostication (EEG reactivity) and identification of epileptiform transients in this relatively small sample of comatose survivors of CA. This finding has an important practical implication, especially for centers where EEG resources are limited.

## Introduction

Cardiac arrest (CA) represents one of the most frequent admission diagnoses for coma, and bears a considerable risk for poor outcome. In recent years, therapeutic hypothermia (TH) has been progressively implemented [1,2], leading to an improvement of functional outcome [3]. In order to distinguish as reliably and timely as possible between patients who will benefit from maximal care and those who will not recover, clinical findings, electroencephalogram (EEG), somatosensory evoked potential (SSEP) and biological parameters (such as neuron-specific enolase (NSE)) have been extensively studied before [4] and since [5,6] TH implementation. The EEG has a central role in this setting, as it allows a noninvasive assessment of brain function and a quantification of the extent of neurological damage [7], as well as the identification of nonconvulsive status epilepticus [8,9].

The International Federation of Clinical Neurophysiology (IFCN) recommends that the usual technical EEG requirements should also been applied when assessing comatose patients [10]. The importance of ruling out confounding factors is emphasized, but the optimal duration of the EEG evaluation is not stated. Continuous EEG monitoring (cEEG) in the ICU has been consistently developed and studied in the last decade [11]; however, unsolved issues remain, particularly regarding the optimal duration of EEG, and whether intermittent standard EEG (sEEG) yields comparable performances with cEEG. This is of particular importance since cEEG is both resource and time consuming. In fact, in the setting of coma after CA where EEG background reactivity represents one of the most important aspects of prognostication [7,12,13], several hours of cEEG recording without stimulation might not necessarily increase the accuracy of outcome prediction. A recent study on the prognostic role of cEEG has also raised this highly relevant issue [14].

In view of these practical aspects, especially in the many centers with limited EEG resources, this issue is of broad interest. We therefore undertook this analysis in comatose survivors of CA aiming to compare the clinical yield of cEEG to that of two 20-minute sEEG recordings, performed at two time points, during TH and after rewarming in normothermic conditions.

## Materials and methods

### Patients and general management

We studied a prospective cohort of consecutive comatose adults treated with TH after a cardiac arrest in our 32-bed multidisciplinary intensive care unit (ICU) between April 2009 and April 2010 (12 months). All patients had had a cEEG at that time (as part of a previously published study protocol) [15]. The need for informed consent was waived due to the observational nature of the study. This study received the full approval of our local ethics commission: the 'Commission cantonal d'éthique de la recherche sur l'être humain'. TH was started immediately in the emergency room and continued in the ICU. Patients were cooled to 33 ± 1°C for 24 hours using ice packs, cold infusions, and an automated surface-cooling device (Arctic Sun, Medivance, Louisville, CO, USA), and given a standardized sedation - analgesia with midazolam (0.1 mg/kg/hr) and fentanyl (1.5 μg/kg/hr). Vecuronium was used to prevent shivering. Sedation was weaned after passive rewarming at 35°C. Patients diagnosed with brain death after rewarming were excluded from the analysis. Withdrawal of care was decided after an interdisciplinary discussion, based on clinical and electrophysiological findings (but not including results of the hypothermic EEG, and normothermic EEG reactivity), as recently described [12].

### EEG and clinical assessment

Long-term video-cEEG (Viasys Neurocare, Madison, WI, USA) was started as soon as possible after ICU admission and during TH (due to limited EEG technologist availability overnight), using 9 to 21 electrodes arranged according to the international 10 to 20 system, and maintained for up to at least 6 hours after rewarming beyond 35°C. Reactivity evaluation was performed with auditory and noxious stimulations by a physician at the bedside during TH and after rewarming. EEGs were interpreted by certified electroencephalographers at the bedside and *post hoc*, using bipolar and referential montages, with a filters setting of 0.5 and 70 Hz, and a notch filter (50 Hz).

All patients were routinely examined after rewarming, off sedation; brainstem reflexes (pupillary, oculocephalic, corneal) and motor reactivity to painful stimulation were assessed. Bilateral median nerve SSEP recordings and serum NSE dosages were also performed but are not analyzed in the present study.

### Data collection

Demographics, cardiac arrest (CA) etiology, duration of CA (defined as the time from collapse to the return of a spontaneous circulation (ROSC)) were prospectively collected.

Brainstem reflexes were categorized as all present or absent. Motor response to pain was categorized as no response or extension posturing vs. flexor response or better.

EEG findings were categorized as previously described [12]: (1) EEG background reactivity, defined as a clear and reproducible change in background frequency or amplitude following auditory or noxious stimulation, excluding stimulus-induced rhythmic, periodic or ictal discharges (SIRPIDs) and muscle artifacts; (2) spontaneous discontinuous (burst-suppression) pattern, defined as an EEG background interrupted by very low-voltage (<5 μV) periods for >10% of the recording; (3) epileptiform activity, defined as any periodic or rhythmic spikes, sharp waves, spike-waves, or rhythmic waves evolving in amplitude, frequency, or field.

To compare cEEG to sEEG performed during TH and after rewarming, two extracts of 20 consecutive minutes from the original cEEG were prepared by the first author (VA), in order to include the first reactivity test during TH (adding 10 minutes before and 10 after), and the first test in normothermic conditions. These EEG extracts were reviewed by two experienced electroencephalographers (AOR and ASM), blinded to patient outcome. In cases of discordant categorization, a consensus decision was reached. Continuous recordings were assessed through the detailed reports and, if needed, by looking at the original EEG data. Agreement between cEEG and sEEG was assessed for background reactivity, continuity, and the presence of epileptiform activity, both during TH and in normothermic conditions. Perfect agreement between cEEG and sEEG was defined by agreement for the three items at both time points.

Outcome was assessed using in-hospital mortality and the Glasgow-Pittsburgh Cerebral Performance Categories (CPC) at three months, where a good outcome is defined by CPC 1 (no impairment) or 2 (moderate impairment) [16].

### Statistical analysis

Data were analyzed using Stata 11.1 (StataCorp, College Station, Texas, USA) and described using frequencies, median and range. Groups were compared using the Wilcoxon rank sum test. Significance was assumed at *P *< 0.05. 95% confidence intervals (CI) were calculated assuming a binomial distribution. Concordance was calculated using Cohen's Kappa.

## Results

During the studied period, 34 cEEG were performed for comatose patients surviving a CA and undergoing TH. Table 1 illustrates baseline clinical characteristics and outcome. Most patients were male and suffered from ventricular fibrillation. There were 55.8% (19/34) survivors at hospital discharge, and the majority of them (14/19) reached a good neurological outcome (CPC of 1 to 2) at three months. EEG data are summarized in Table 2. Of note, of the 15 patients who died, 12 (75%) showed a nonreactive pattern and 11 (73%) prolonged discontinuous activity. The median time from CA to the start of cEEG was 12.5 hours (range: 1 to 23), the median time between CA and the first reactivity assessment was 17.4 hours (range: 4.75 to 25), and the median duration of cEEG was 26 hours (range: 19 to 66).

**Table 1:** Demographics, etiology, clinical characteristics and outcome.

Patient characteristics	*n *= 34
Age (mean)(SD; range)	61 (13.2; 32 - 84)

Female	9 (27%)

CA of cardiac etiology	27 (79%)

	

Initial cardiac arrest rhythm	

Asytole	3 (8.8%)

Pulseless electric activity	8 (23.5%)

Ventricular fibrillation	23 (67.65%)

	

Time to ROSC (min), mean (SD; range)	27.8 (30.4; 5 - 180)

	

Clinical characteristics at rewarming:	

Presence of all brainstem reflexes	20 (58.8%)

Flexor motor response or better	16 (47.1%)

Myoclonus	7 (20.6%)

	

In-hospital survivors	19 (55.9%)

Vegetative state	0 (0%)

Minimal conscious state	1 (5.3%)

Awakening	18 (94.7%)

	

CPC at 3 months	

1	8 (23.5%)

2	6 (17.65%)

3	5 (14.7%)

4	0 (0%)

5	15 (44.1%)

CA, cardiac arrest; CPC, Cerebral Performance Category; ROSC, return of spontaneous circulation; SD, standard deviation.

**Table 2:** Electroencephalography (EEG) characteristics.

EEG characteristics					
***n *= 34**					
**Hypothermia: **	***Continuous EEG***	***Standard EEG***	***Agreement***	***95% CI*** ***(confidence*** ***interval)***	***Concordance (Cohen's kappa)***
**Discontinuous background/flat **	12 (35.3%)	13 (38.2%)*	33 (97.1%)	84.6 - 99.9	
**Nonreactive EEG background**	13 (38.2%)	14 (41.2%)*	33 (97.1%)	84.6 - 99.9	
**Epileptiform discharges**	8 (23.5%)	6 (17.7%)**	32 (94.1%)	80.3 - 99.2	0.83
					
					
**Normothermia:**	***Continuous EEG***	***Standard EEG***	***Agreement***	***95% CI***	
**Discontinuous background/flat **	12 (35.3%)	12 (35.3%)	34 (100%)	89.7 - 100***	
**Nonreactive EEG background **	13 (38.2%)	13 (38.2%)	34 (100%)	89.7 - 100***	
**Epileptiform discharges **	9 (26.4%)	9 (26.4%)	34 (100%)	89.7 - 100***	1

*In the same patient, the EEG started with a nonreactive burst-suppression pattern, which evolved during therapeutic hypothermia to a continuous and reactive trace. Continuous EEG monitoring was started 6 h after cardiac arrest; **epileptiform discharges were missed in two patients. One had very rare spikes and waves missed with standard EEG, and the other became epileptiform only 12 hours after cEEG start; his EEG was started 12 h after cardiac arrest; ***one-sided 97.5% confidence interval

During TH, as compared to cEEG data, the interpretation of sEEG recordings labeled one additional patient with discontinuous activity and a nonreactive background. His cEEG was started 6 hours after his CA; the initially nonreactive discontinuous pattern (overlapping with the first reactivity testing) gradually evolved after some hours to a continuous and eventually (after an additional 15 hours, still under TH) reactive background. Thus, the agreement between cEEG and sEEG during TH was 97.1% (95% CI: 84.6 to 99.9%) for discontinuity and reactivity evaluation. Regarding epileptiform activity, sEEG identified six traces, as compared to eight diagnosed with cEEG. Of these two additional patients identified with cEEG, one had very rare spikes and waves missed on the 20-min sEEG, and the other developed an epileptiform EEG 12 hours after the start of cEEG (at 24 hours from CA). The agreement for epileptiform activity was therefore 94.1% (95% CI 80.3 to 99.2%). We found a very good concordance between two sEEG and cEEG (kappa = 0.83) during therapeutic hypothermia.

In normothermic conditions, the agreement between cEEG and sEEG was 100% (95% CI: 89.7 to 100%) for the three items. Globally, perfect concordance at both time points was obtained for 31/34 patients (91.2%; 95% CI: 76.3 to 98.1%). The concordance between sEEG and cEEG was perfect during normothermic conditions (kappa = 1).

The median time between CA and the first EEG background reactivity testing was 18 hours (range: 4.75 to 25) for patients with a perfect agreement between cEEG and sEEG and 10 hours (range: 5.75 to 10.5) for the three patients with discordant EEG (*P *= 0.02, Wilcoxon rank sum test).

## Discussion

The principal finding of this study is that two repeated standard EEG evaluations with reactivity testing at both time points, during hypothermia and normothermia, seem as efficient as continuous EEG for the management of survivors of CA.

We found a perfect concordance between sEEG and cEEG in normothermic conditions, while sEEG and cEEG were discordant in three situations during the hypothermic period. In one patient, the agreement was not perfect for background discontinuity and reactivity: the initial pattern evolved over hours during TH. The cEEG was started very early: EEG background reactivity was tested only 5.75 hours after CA, suggesting that a too timely EEG background evaluation can underestimate the recovery potential. Indeed several predictors, both clinical and electrophysiological, have been shown to become more accurate with the increase of time between CA and evaluation [17]; it is also known from the pre-hypothermia era that even an isoelectric EEG background during the 12 first hours after CA does not exclude a full clinical recovery [18]. This time delay is of practical importance, as a nonreactive EEG background represents a robust predictor of poor outcome after CA and TH [7]. Moreover, sedation may also influence the EEG background in early TH, but in our experience it normally does not abolish background reactivity. We therefore believe that early changes on EEG are mostly due to acute anoxia.

Standard EEG also missed two patients with epileptiform discharges during TH. The timing of EEG recording seems, again, crucial for this aspect. One patient showed epileptiform discharges (but no seizures) only after 12 hours of recording, which were not seen on the initial sEEG performed 10.5 hours after CA. As recently shown, the mean onset of epileptiform activity after CA is 17 hours [19]: in that study, the majority of patients that suffered from postanoxic seizures presented evident epileptiform activity during the first hour of EEG monitoring. The only patient who had postanoxic seizures but did not show epileptiform transients during the first hour of EEG monitoring had his cEEG started 5 hours after CA. Another study found earlier appearance of nonconvulsive postanoxic status epilepticus [20]. Indeed, in that analysis, the median interval between CA and the EEG was 9 hours; 25% of the records showed epileptiform transients at the beginning of cEEG, and around half of the status epilepticus patterns were present within the first 8 hours of recording. Given the data of these two studies [19,20] and our findings, a delay of 9 to 12 hours between CA and the first EEG assessment appears a reasonable compromise. In another study investigating the yield of EEG monitoring in an unselected hospital population of patients who underwent cEEG monitoring, the absence of epileptiform activity in the first 30 minutes of recording rendered subsequent seizures extremely unlikely. However, that cohort included a wide range of etiologies, and only 12% had hypoxic brain injury; latencies between brain injury and cEEG start were not provided. Interestingly, seizures seen within the first 30 minutes of monitoring were more frequent in the subgroups of patients suffering from hypoxic-ischemic encephalopathy after CA, toxico-metabolic encephalopathy, and unexplained altered mental status as compared to other etiologies of impaired consciousness. The frequency of epileptiform discharges may also represent a critical issue: the other patient with missed epileptiform patterns in our study had only very rare, isolated spikes and waves that escaped detection on the 20-minute sEEG. Isolated, nonperiodic epileptiform discharges represented only 21% of the epileptiform EEG patterns in one series [19] and the majority of patients with documented seizures, in fact, presented periodic discharges during the first cEEG-monitored hour. In another study, all patients with electrical status epilepticus presented with continuous discharges [9]. It seems, therefore, that isolated epileptiform discharges are found in a very small proportion of CA survivors, as opposed to periodic elements.

The timing of EEG evaluation appears crucial for background reactivity evaluation and for epileptiform elements identification: there was a significant difference in the delay between CA and EEG reactivity assessment for perfectly concordant evaluation (17.75 hours) versus discordant findings (10 hours).

Our study has several limitations. We used retrospectively 'produced' sEEG obtained from cEEG data, but this is the only way to use the same patient as its own control. Some authors were involved in the treatment of those patients; however, the recordings were rendered anonymous and the delay between data acquisition and the present study was at least two years for every patient. The sample size is relatively small, but confidence intervals are narrow (since the proportions appear robust) and concordance is very good to optimal, thereby reinforcing our main finding. We reviewed sEEG and compared them with the cEEG report information. Because the same standardized report system (including continuity, reactivity and epileptiform discharges) is used since the first recording of the study (under the supervision of AOR), we do not believe that interobserver variation plays a major role in this study. We chose to compare cEEG to two separate sEEGs, since, on the one side, there is increasing evidence that recordings performed during TH, particularly regarding background reactivity, are strong prognosticators of outcome [7], and, on the other side, a multimodal assessment is advocated for outcome prognostication during the post-TH normothermic period, our finding of a perfect agreement in normothermia is thus very reassuring. Intermittent EEG evaluations may delay postanoxic status epilepticus identification for some hours, potentially influencing the outcome. However, only a small subgroup of postanoxic status epilepticus showing a particular clinical profile may ultimately have a good outcome [21] and recent data does not support any clinical benefit from seizure detection and treatment during TH [14]. Another concern is that time to EEG initiation was not uniform, possibly influencing some results. Finally, our study focuses on a particular clinical setting and particular hypothermia protocol, and these findings should not be automatically extended to other etiologies of critically ill patients. Indeed, it has been repetitively shown that cEEG proves important for the management of comatose patients with subarachnoid [22] and intracerebral hemorrhage [23], or traumatic brain injury.

## Conclusions

In conclusion, standard EEGs with background reactivity evaluation performed at two separate time points, during therapeutic hypothermia and normothermia, seem to be as efficient as continuous EEG monitoring in the setting of coma after CA. In order to optimize the performance of this approach, it seems reasonable that the first EEG during hypothermia is performed after at least 9 to 12 hours after CA. The findings reported in the present study are novel and have, in our view, an important implication in clinical practice, particularly for centers managing these patients without the resources to provide cEEG to every subject. Of course, these preliminary data need to be confirmed on a larger cohort. Our results are in line with another recent study, approaching this issue from another angle, which suggests that the management of these patients did not change since routine cEEG implementation, whereas the costs increased [24]. While cEEG represents the investigation of choice in case of seizures or status epilepticus identification, particularly in normothermia, and is of invaluable utility in order to understand the complex and dynamic process of brain anoxia, the current study offers practical data to optimize the yield of EEG recordings in patients suffering from this dramatic neurologic condition. Further analyses of repeated sEEG in other etiologies may be warranted to assess the role of this approach as compared to cEEG.

## Key messages

• Standard intermittent EEG has comparable performance to continuous EEG both for variables important for outcome prognostication (EEG reactivity) and identification of epileptiform transients in comatose survivors of CA.

• Too early EEG background evaluation can underestimate the recovery potential

• Continuous EEG should remain the investigation of choice in case of seizures or status epilepticus identification.

## Abbreviations

CA: cardiac arrest; cEEG: continuous electroencephalography; CI: confidence interval; EEG: electroencephalography; NSE: neuron-specific enolase; sEEG: standard electroencephalography; SSEP: somatosensory evoked potential; TH: therapeutic hypothermia.

## Competing interests

Vincent Alvarez has nothing to disclose. Alba Sierra-Marcos has nothing to disclose. Mauro Oddo is supported by grants from the Swiss National Science Foundation (320030_138191) and the European Society of Intensive Care Medicine (ECCRN Clinical Research Award). Andrea O. Rossetti is supported by a grant from the Swiss National Science Foundation (CR32I3_143780), and received research support from UCB and GSK.

## Authors' contributions

VA designed the study, was responsible for data acquisition and analysis, performed the statistical analysis and drafted the manuscript. ASM analyzed the data and drafted the manuscript. MO was responsible for data acquisition and drafted the manuscript. AOR designed the study, was responsible for data acquisition and analysis, performed the statistical analysis, drafted the manuscript and supervised the study. All authors read and approved the final manuscript.
